# Comparison between the treatment area of electrode used for radiofrequency ablation of liver cancer focusing on 15G cooled-tip and CWT electrode

**DOI:** 10.12669/pjms.323.8538

**Published:** 2016

**Authors:** Hyun-Jin Kim, Hae-Kag Lee, Jae-Hwan Cho

**Affiliations:** 1 Hyun-Jin Kim, Department of Radiology, Asan Medical Center, Seoul Republic of Korea; 2 Hae-Kag Lee, Department of Computer Science and Engineering, Soonchunhyang University, Asan, Republic of Korea; 3 Jae-Hwan Cho, Department of Radiological Technology, Ansan College, Ansan, Republic of Korea

**Keywords:** Hepatocellular carcinoma (HCC), Radiofrequency ablation, Cool Wet-tip (CWT), 15G

## Abstract

**Objectives:**

To analyze the comparison between the treatment area of 15Gage internally cooled electrodes and 17 Gage Cool Wet-tip(CWT) electrodes. They are manufactured to broaden treatment area of the tumor in the radiofrequency ablation of hepatocellular carcinoma(HCC).

**Methods:**

The study was designed for 62 patients with a mean age of 61, ranging from 44 to 87 years. The sample comprised of patients who used 15 G internally cooled electrodes and 17 G CWT electrodes respectively. Computed tomography (CT) images obtained after the procedure were observed, however, for the ablation lesion, the volume was determined by measuring complete necrotic tissue that did not contrast enhancement in the image.

**Results:**

The treatment area of the tumor after radiofrequency ablation was 17.26±6.02 in the CWT, which was bigger than 15G. The treatment area ratio of the treatment before or after was significant at 581.85±339.56 in the CWT. After radiofrequency ablation, the treatment area got bigger, as 15G electrodes went toward CWT electrodes. Treatment area per electrode was 1.34 times higher in CWT than in 15G while the treatment area ratio of the treatment before or after was 1.001 times higher in the CWT than 15G.

**Conclusions:**

Ablation is more common for the safety margin in stable tumor and CWT type electrodes that can make larger ablation to reduce the number of times ablation is required for residual tumor and it decreases recurrence, ablation time and reoperation. Therefore it is considered t useful to reduce patients’ pain.

## INTRODUCTION

Hepatocellular carcinoma (HCC) is one of the major cancers worldwide, with an increased prevalence in the West.^1^-^4^ The survival rate among liver cancer patients from 2006 to 2010 was at 26.7%. However, the survival rate had improved twice as compared to 10 years ago. On the contrary, there was poor prognosis in HCC in comparison to other forms of cancer.^5^ Radical treatments among the patients with HCC are topical therapies such as liver transplantation, surgical resection, radiofrequency ablation and ethanol injection. There have been many studies on the safety and treatment results of radiofrequency ablation over the last 10 years.

The radiofrequency ablation has been widely used in the treatment for early HCC and its efficacy is being enhanced with the introduction of new technology. When high frequency current of 460-500 kHz is applied to electrodes that are inserted inside the tumor, molecular motion is induced and frictional heat is generated by friction heat. Radiofrequency ablation generates this frictional heat in tumor at the surrounding tissues and induces tumor necrosis. In general, tumor tissue brings irreversible damage caused by the destruction of the cell and protein denaturation if more than 45-50ºC temperature remains for several minutes. High frequency electrodes that are used to treat liver cancer have various forms and methods that are used according to each user needs. Among these electrodes, the electrode of 17 G (CWT) type that increases treatment area by the emission of saline through the side hole of the electrode tip is widely used. In recent years, 15 Gage electrode of internally cooled electrode type that increases treatment area by increasing the inner diameter of the electrode has been newly developed^6^

The purpose of radiofrequency ablation is to enhance the survival of the patient by causing much bigger treatment area about tumor and tissue necrosis of a fixed size, so the selection of electrodes that influence safe treatment area is considered to be very important. Thus, the treatment area of two electrodes was evaluated. The purpose of this study was to compare and analyze treatment area between 15Gage single cool tip that is used for radiofrequency ablation and cool wet-tip.

## METHODS

Retrospective analysis was done for the 62 patients (mean age: 61years, range: 44-87 years) that used 3 cm of 15 G internally cooled electrodes and 3cm of 17G Cool Wet-tip (CWT) electrodes. Moreover, patients with one lesion and tumor size of less than 3cm and who underwent ablation one time, among patients who underwent radiofrequency ablation for primary hepatocellular carcinoma (HCC) were reviewed as well. All participants signed a written informed consent Form approved by the Institutional Review Board at Hallym University of Graduate Studies.

Electrodes that were used for the treatment were 17G CWT and 15G electrodes. In order to derive the electrode to the lesion, ultrasound device (LOGIQ E9, GE Company, USA) and convex probe of 1-5MHz was used. In addition, M-224 and RFP-100 high frequency generator and cooling pump were also used. Furthermore, adaptor cable, power able (for Generator, for Pump), cable for PAD connection, cable for PAD connection reuse, Inflow tube, Outflow tube, and Ground PAD were used. Procedure time refers to the time that takes to completely ablate hepatocellular carcinoma (HCC) and both electrodes were only used for the patients who finished treatment after 12-minute ablation. Arterial phase, venous phase, and 3-minute delayed phase obtained after pre-contrast and intravenous contrast material injection, by using light speed VCT (GE, USA), CT (Somatome, SIEMENS, Germany) for the verification of treatment area after procedure were observed. After CT, complete necrotic tissue that did not contrast enhancement from the images obtained through multilevel reconstruction was measured in order to measure the size of the lesion. Volume was found by measuring X and Y-axis value from the axial scan of CT images and Z-axis value from Coronal scan. The volume of ablation lesion was obtained using the formula of ‘Volume=b (A×B×C)/6 by measuring the three-axis length from the central axis. In the formula, A, B, and C means length of three-axis of ablation lesion. For statistical analysis, a T-test was done for the average comparison of treatment area ratio of the area before and after the tumor treatment and it was analyzed by correlation coefficient. The treatment area of two electrodes was compared by performing regression analysis and logistic regression analysis of the relevant variables. Statistical analysis was done with SPSS ver.18.0 software and the statistical significance p value of each output was defined as 0.05(SPSS Inc. Chicago, IL, USA).

## RESULTS

The average comparison of pre-treatment tumor volume, post-treatment tumor volume and treatment area ratio (before and after treatment) of 15G and CWT by electrode are shown in Table-I. The tumor treatment area after radiofrequency ablation was larger in CWT (17.26±6.02) than 15G and treatment area ratio before and after the treatment was shown to be great in CWT (581.85±339.56) (p<0.05).

**Table-I:** The average comparison of pre-treatment tumor volume, post-treatment tumor volume and treatment area ratio of 15 gage and cool wet-tip.

*Division*	*Classification*	*Average*
Pretreatment volume	15G	3.55 ±2.32
	CWT	3.74±2.06
Post-treatment volume	15G	11.53±3.46
	CWT	17.26±6.02
Treatment area ratio	15G	456.97±284.08
	CWT	581.85±339.56

*Note:* G; gage, CWT; cool wet-tip

The results of the correlation analysis of pre-treatment tumor volume, post-treatment tumor volume and treatment area ratio by each electrode are shown in Table-II. There was no correlation in pretreatment tumor volume by electrode, but post-treatment tumor volume had a positive correlation (0.51) and treatment area ratio before and after treatment had a positive correlation (0.20) (p<0.05). The results of the scatter plot graph by each electrode after radiofrequency ablation for related factors, after Pearson correlation analysis showed that R-squared (R²) was 0.257. Thus, there was a correlation between the treatment area after radiofrequency ablation with 15 G electrode and CWT electrode. In other words, as 15 G electrode went towards CWT electrode, treatment area got larger.

**Table-II:** Correlation analysis of pretreatment tumor volume, post-treatment-tumor-volume and treatment area ratio before and after treatment by electrode.

*Division*		*Classification*	*Pretreatment volume*	*Post-treatment volume*	*Treatment area ratio*
Classification	Correlation coefficeint	1.00	0.04	0.51	0.20
Pretreatment volume	Correlation coefficient	0.04	1.00	0.06	-0.66
Post-treatment Volume	Correlation coefficient	0.51	0.06	1.00	0.53
Treatment area ratio	Correlation coefficient	0.20	-0.66	0.53	1.00

The scatter plot graph results of the treatment area ratios of radiofrequency ablation before or after showed that R-squared (R²) was 0.039, the statistical data demonstrated that there was no correlation between treatment area ratio before and after treatment when using 15 G electrodes and CWT electrode because the Pearson correlation (p> 0.05) was greater than 005 at 0.039. In other words, the treatment area ratio in the use of 15G had no direct effect in the treatment area ratio that uses CWT electrodes. The results of the regression analysis showed that the constant value by variables of tumor area after radiofrequency ablation by electrodes and standard error were 5.74±0.88.

The treatment area ratio before or after treatment was 124.88±56.22. As a result, tumor area and treatment area ratio before and after radiofrequency ablation treatment, demonstrate the increase in CWT than 15 G (Table-III) (p<0.05). The results of logistic regression analysis of relevant variables are given in Table-IV. The post-treatment area by electrodes was 1.34 times higher in CWT than 15 G and treatment area ratio either before or after was 1.001 times higher in CWT than 15 G (Table-IV) (p<0.05).

**Table-III:** Regression analysis of variables.

*variables*	*B*	*SE*
Post-treatment volume	5.74	0.882
Treatment area ratios	124.889	56.226

**Table-IV:** Logistic regression analysis of variables.

*Classification*	*B*	*S.E*	*OR*	*95% CI*	

				*Lower*	*Upper*
Post-treatment volume	0.295	0.060	1.343	1.194	1.510
Treatment area ratios	0.001	0.001	1.001	1.000	1.002

*Note:* OR; odds ratio, 95% CI; 95% confidence interval

## DISCUSSION

Radiofrequency ablation is used widely for non-surgical treatments when surgery is unbearable to patients with malignant liver tumors. Moreover, it is used when the location of the lesion does not permit for a surgical operation.^7^ Electrodes that are used for the radiofrequency ablation multipolar internally cooled Cluster, CWT-type electrode, monopolar internally cooled cool-tip, etc, but CWT-type electrode and cool-tip type electrode is being widely used now.^8^ McGhana JP et al. made a 3-cm part that is not isolated at the end of needle with cool-tip type electrode which consists of a single needle. He announced that he could prevent the burning or boiling of surrounding tissue due to the overheating of the electrode and broaden treatment area by placing cold normal saline solution inside the needle for its circulation.^9^

Kim JH et al. published that ablation area was larger than conventional methods by preventing any possible hydrocarbons that in adjacent tissue of electrode by using the Cool-wet tip (CWT) made by complexly applying coolant circulation system and the discharge of normal saline solution.^10^,^11^ In this study, the treatment area of tumor after radiofrequency ablation was larger in CWT than 15 G and the treatment area ratio before and after treatment was also larger in CWT. It appeared that R-squared of scatter plot graph by each electrode after Pearson correlation analysis was 0.257, however s the 15 G electrode went toward CWT electrode, treatment area got wider. It was shown that R-squared of scatter plot graph about treatment area ratio before and after radiofrequency ablation was 0.039. Nonetheless, the 15 G electrode went toward CWT electrode, treatment area ratio got wider.

In addition, regression analysis results showed that tumor area and treatment area ratio before and after radiofrequency ablation treatment increased in CWT than 15 G. The results of logistic regression analysis also showed that post-treatment area was 1.34 times higher. Conversely, the treatment area ratio before and after treatment was 1.001 times higher in CWT than 15 G.

### Limitations of the study

The limitation of this study was that there might be difference in volume according to observer’s view through simple visual measurement on CT images and pathological findings in the calculation of the volume of ablation lesions.

## CONCLUSIONS

As per results of this study, it was found that the size of treatment area with radiofrequency ablation using CWT electrode was ablated more broadly than that using 15 G internally cooled electrode. Ablation is generally used more than once for the safety margin of stable tumor. Thus, it is considered that CWT-type electrode that makes a larger ablation lesion is useful in patient’s pain reduction because it reduces the number of ablation and residual tumor, which shortens ablation time and reduces reoperation.
